# Adherence to adjuvant endocrine therapy after breast cancer in Sweden – a nationwide cohort study in 1-, 3- and 5-year survivors with a focus on regional differences

**DOI:** 10.2340/1651-226X.2024.40575

**Published:** 2024-11-24

**Authors:** Anna de Jong, Anna Von Wachenfeldt, Lennarth Nyström, Anne Andersson

**Affiliations:** a Department of Diagnostics and Intervention, Oncology, Umeå University, Umeå, Sweden; b Department of Clinical Science and Education, Karolinska Institute, Södersjukhuset, Stockholm, Sweden; c Department of Epidemiology and Global Health, Umeå University, Umeå, Sweden

**Keywords:** Adherence, early breast cancer, endocrine therapy, adjuvant treatment

## Abstract

**Background and purpose:**

Adjuvant endocrine treatment (AET) is crucial in early oestrogen receptor (ER)-positive breast cancer (BC), providing reduced recurrence rate and increased overall survival. The aim of this study was to estimate AET adherence rates by age at diagnosis and region in Sweden.

**Patients and methods:**

In total, 10,422 women diagnosed with ER-positive BC in 2008–2010 were identified in the Swedish National BC Registry. Information on prescriptions and dispensation of AET was gathered through record linkage to the Swedish Prescription Registry. 1, 3- and 5-year medication possession ratios (MPRs) were calculated. Good adherence was set as MPR ≥ 80%.

**Results:**

The 1-, 3- and 5-year AET age-adjusted adherence rates were 94.4, 87.6 and 81.6%, respectively. The 1-, 3- and 5- year adherence rate was significantly highest in the South region (96.2, 90.5 and 86.2%). Regions with an oncologic clinic had higher adherence rate than regions without, 82.8% versus 75.5% at 5-year FU. Women at age 40–64 years (95.6, 89.9 and 84.1%) and 65–74 years at diagnosis (95.7, 89.5 and 84.6%) had significantly higher adherence rate than women ≥ 75 years at diagnosis (89.1, 79.2 and 68.3%).

**Interpretations:**

Despite guidelines being national, there were significant differences in adherence between regions in Sweden. As the largest differences were between age groups invited and not invited to mammography screening intervention should focus on women < 40 and ≥ 75 years at diagnosis. Further studies are needed to find strategies to increase overall adherence to AET in early BC.

## Introduction

Breast cancer (BC) is the most common type of cancer in women worldwide. In Sweden, 29% of newly diagnosed cancers in women in 2018 were BCs (10,063 new cases in 7,858 women). For oestrogen receptor (ER)-positive tumours, adjuvant endocrine treatment (AET) with tamoxifen (TAM) or aromatase inhibitors (AIs) is offered for at least 5 years. AET decreases the oestrogen-stimulated cell division in ER-positive BC and thereby reduces BC recurrence and BC mortality, regardless of age at diagnosis or other adjuvant treatment [1]. TAM blocks the oestrogen receptor, whereas AIs inhibit the conversion of androgens into estrone and oestradiol. AIs are preferably used in postmenopausal women with no oestrogen production in the ovaries. As oestrogen production occurs in the functioning ovaries of premenopausal women, ovarian suppression with gonadotropin-releasing hormone (GNRH) is necessary when AIs are used as AET in this group. Both TAM and AIs are effective in reducing recurrence and mortality in BC [2].

Treatment recommendations for AET vary with ER-, tumour- and nodal status [3]. Recent studies suggest that the positive effect of AET can be increased with an extended treatment duration (up to 10 years) with TAM and/or AI, resulting in an increased reduction of recurrence and mortality in BC [1, 4, 5]. AET is associated with adverse effects due to oestrogen deficiency, for example, musculoskeletal ache/pain, hot flashes, fatigue, dryness of the vaginal mucosa and sexual dysfunction. In younger fertile women, AET can interfere with the wish of pregnancy. Most patients experience adverse effects, which can have a negative impact on quality of life [6, 7]. Previous national and international studies have revealed that adherence to AET is lower than expected and suboptimal [5, 8–11].

Medical possession ratio (MPR) is a well-known estimate of adherence of medical treatment and an MPR of ≥ 80% is often used as a cut-off for good adherence [9, 11–13]. Previous studies of AET adherence in Sweden have been performed in one or a few geographical areas. The 3-year adherence of 1,741 women diagnosed with BC in 2005 in the Stockholm-Gotland and Uppsala-Örebro regions was 69% [9]. In another study, the 1-, 3-, and 5-year adherence rates of 3,395 women diagnosed in Stockholm-Gotland in 2005–2008 were 86, 64 and 46%, respectively [5]. Furthermore, the 3- and 5-year adherence rates of 488 women in the Jönköping region in 2009–2012 were 91 and 92%, respectively [12]. Currently, there are neither estimates of adherence to AET for early BC nationwide in Sweden nor comparisons between all regions in Sweden. Previous international studies on adherence have shown a large variation in 5-year adherence, ranging from 46% to 88% [7, 10, 14–18]. Depending on which method that is used for when measuring adherence, numbers can differ, why these number might not be directly comparable.

In Sweden, BC is treated in accordance with national guidelines. However, these guidelines were previously supplemented with regional guidelines based on, among other things, local access to oncology and surgery specialists. These regional differences may have affected follow-up (FU) routines after treatment, for example, which clinic or caregiver was responsible for the FU.

Sweden is geographical divided into six public health care regions (HCRs) and all HCRs but one is geographical divided further, creating a total of 21 regions. Each region is divided into municipalities (290 in total). The municipalities are categorised into three main groups and nine subgroups depending on number of inhabitants in the municipality and the distance to large cities. Large cities have over 200,000 inhabitants, middle sized towns have 40,000 – 199,999 inhabitants and the smallest towns have less than 40,000 in the urban area of the city. There is at least one oncology clinic in each of the six HCR but since not every hospital and region has an oncology clinic, oncologists or patients must often travel to another hospital or even another region for an oncologic consultation. There are six out of 21 of the regions that lack oncology clinic. Treatment and FU of AET are often administered within a specialty other than oncology, for example, by surgeons or general practitioners.

Estimating adherence to AET nationwide and in regions could reveal subgroups in which targeted efforts should be made to increase adherence and thereby reduce the risk of BC recurrence. Thus, the aim of this study was to study patterns in AET adherence at 1-, 3- and 5 years after the first dispensation, by region and age at diagnosis in women diagnosed with BC in Sweden in 2008–2010.

### Patients and methods

This is a cohort study, based on three population-based registries: the Swedish Cancer Registry (SCR), the Swedish National Breast Cancer Registry (NBCR) and the Swedish Prescription Registry (SPR). All newly diagnosed BCs in Sweden since 1958 have been reported to the SCR. It contains basic information on patient and tumour characteristics. The NBCR was established in 2008 and originated from regional BC registries. It includes clinical information, for example, pre- and post-operative diagnostic data, type of surgery, data on tumour characteristics and planned and given treatment. Coverage is high, at least 95% in both registries [19, 20]. The SPR, established in 2005, gathers data from all pharmacies on prescription dates, dispensing dates, number of packages, defined daily doses and Anatomical Therapeutic Chemical Classification codes (ATC).

Women with primary BC, stage I-III, diagnosed in 2008–2010 in Sweden were identified using the SCR. The cohort was linked to the NBCR to obtain information on tumour and patient characteristics. In women with more than one BC registered, the characteristics of the tumours were reviewed manually and data relating to the tumour with the most severe characteristics were included in the study. Individuals with missing data regarding invasiveness, pathological anatomic diagnosis (PAD), ER-, T- or M-status or known presence of distant metastasis were excluded from the study. N-status did not affect the indication of AET why individuals with missing N-status were included in the study. Patients with small tumours (≤10 mm) without positive nodes were also excluded, since AET was not included in the nationwide treatment recommendations for such patients. Patients with recurrent disease before 5-year follow-up were excluded from the study.

**Figure 1:**
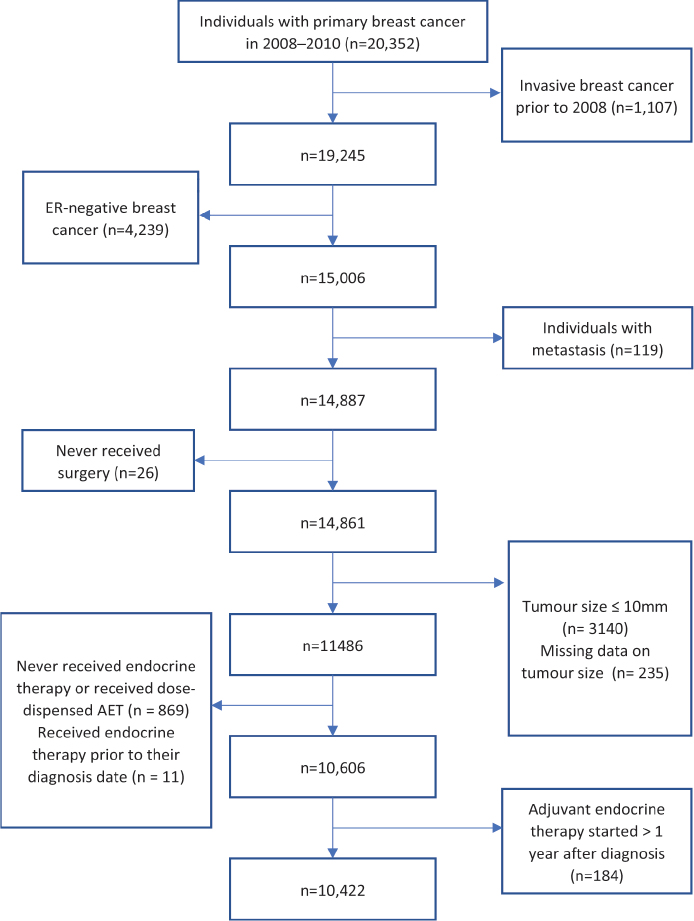
Flowchart of selection process. ER: oestrogen reecptor, AET: adjuvant endocrine treatment

Linkage to the SPR was then performed to obtain data on prescriptions, dispensations, and type of AET in 2005–2016, where AET included tamoxifen and aromatase inhibitors (ATC L02BA01, L02BG03, L02BG04 and L02BG06). Individuals receiving medication through a dispensing system or switching to a dispensing system during the treatment period were excluded from start, since administration of drug was often monitored by another person. ER-positive BC patients who never received an AET prescription were excluded as individuals treated with endocrine treatment with TAM or AI before time of diagnosis for reasons other than BC. Patients deceased at the 1, 3- and/or 5-year FU were excluded from the calculation of AET adherence at that timepoint. The data generation is illustrated in a flowchart (Figure 1).

At least one expedition of AET during the first year was one of the inclusion criteria in the study. Data on all expedition dates, type of AET and numbers of pills at each expedition were retrieved from SPR. The expedited numbers of pills, which is taken once daily, was set to correspond to the same numbers of days on treatment. Adherence was defined as MPR and calculated as the total number of days with supply of any AET divided by the number of days at the 1-, 3- and 5-year FU from date of first expedition of AET. Good adherence for an individual patient was set as MPR ≥ 80%. Overlapping dates for expedition or prescriptions of AET was not registered. The number of pills at each expedition varied over time and between patients. One calendar year was set to 365 days.

The classification of the Swedish Association of Local Authorities and Regions was used to divide municipalities into three main groups (large cities and municipalities near a large city [*n* = 46], medium-sized towns and municipalities near medium-sized towns [*n* = 108] and small towns and rural municipalities [*n* = 136]). Analysis was performed based on patients’ residence at start of AET. Data on migration during FU were not available.

### Statistical analysis

Wald’s statistic was used to calculate 95% confidence intervals (CIs) for the MPR. Logistic regression analysis with odds ratios (ORs) and 95% CIs was used to estimate the likelihood of AET adherence by age at diagnosis, HCR, the presence of oncologic clinic in the region and main municipality groups. Adherence rate by region, presence of oncologic clinic and municipality was adjusted by age. Adherence was also controlled for T- and N-status at diagnosis. IBM SPSS 29.0 was used for the statistical analysis.

The study was approved by the Ethical Review Board in Umeå (Dnr 2016-158-31M).

## Results

In total, 20,352 women with primary BC were identified in the SCR in 2008–2010 and 10,422 remained after exclusion (Figure 1). Mean age at diagnosis was 62.2 years (Range: 23–101). At the 1-, 3- and 5-year FU, 10,280, 9,836 patients and 9,256 patients, respectively, were alive at FU and remained for the statistical analysis of adherence. The characteristics of the patients are presented in Table 1. Adherence to AET was 94.4, 87.7 and 81.7% at 1-, 3- and 5-year FU, respectively (Table 2).

**Table 1:** Tumour characteristics, mean age, and planned adjuvant therapy among women diagnosed with ER-positive primary breast cancer in Sweden 2008–2010 and at 3- and 5-year follow-up after first expedition of AET.

Characteristics	At time for breast cancer diagnosis	Alive at 1-year follow-up	Alive at 3-year follow-up	Alive at 5-year follow-up
Total (*n*)	10,422	10,280	9,836	9,256
Mean age year (range)	62.2 (23–101)	62.0 (23–101)	61.6 (23–101)	61.1 (23–97)
T-status, post-surgery, *n* (%)				
T1	5,766 (55.3)	5,720 (55.6)	5,564 (56.6)	5,377 (58.1)
T2	4,080 (39.1)	4,002 (38.9)	3,766 (38.3)	3,449 (37.3)
T3	576 (5.5)	558 (5.4)	506 (5.1)	430 (4.6)
Unknown/missing (%)	0 (0.0)	0 (0.0)	0 (0.0)	0 (0.0)
N status, post surgery, *n* (%)				
N0	6,204 (59.5)	6,133 (59.7)	5,937 (60.4)	5,667 (61.2)
N1	2,926 (28.1)	2,893 (28.1)	2,782 (28.3)	2,632 (28.4)
N2	902 (8.7)	877 (8,5)	802 (8.2)	716 (7.7)
N3	375 (3.6)	365 (3,6)	303 (3.1)	232 (2.5)
Unknown/missing	15 (0.1)	12 (0.1)	12 (0.1)	9 (0.1)
Detection through screening, *n* (%)				
Yes	4,653 (44.6)	4,630 (45.0)	4,525 (46.0)	4,378 (47.3)
No	5,713 (54.8)	5,594 (54.4)	5,257 (53.4)	4,831 (52.2)
Unknown/missing	56 (0.5)	56 (0.5)	54 (0.5)	47 (0.5)
Receptor status, *n* (%)				
ER-positive	10,422 (100.0)	10,280 (100.0)	9,839 (100.0)	9,258 (100.0)
HER2 amplified	1,038 (10)	1,022 (9.9)	972 (9.9)	903 (9.8)
Planned adjuvant therapy				
Chemotherapy (%)	3,374 (32.4)	3,349 (32.6)	3,232 (32.9)	3,065 (33.1)
Radiotherapy (%)	7,469 (71.7)	7,395 (71.9)	7,139 (72.6)	6,796 (73.4)
Trastuzumab (%)	670 (6.4)	668 (6.5)	646 (6.6)	619 (6.7)

All women received adjuvant endocrine treatment.

T: tumour size; N: Lymph nodes; ER: oestrogen receptor; HER2: human epidermal growth factor receptor 2.

**Table 2:** Likelihood of adherence to adjuvant endocrine treatment. Bivariate logistic regression analysis (odds ratio [OR] and 95% confidence interval [CI]) at 1-, 3- and 5-year follow-up by age group.

	Total	At 1-year follow-up					At 3-year follow-up					At 5-year follow-up				
		Alive *n*	Adherent to treatment *n* (%)	95% CI	OR	95% CI	Alive, n	Adherent to treatment *n* (%)	95% CI	OR	95% CI	Alive n	Adherent to treatment *n* (%)	95% CI	OR	95% CI
All	10,422	10,280	9,707 (94.4)	94.0–94.9	-	-	9,849	8,625 (87.7)	87.0–88.3	-	-	9,271	7,559 (81.7)	80.9–82.4	-	-
–39	349	346	326 (94.2)	91.2–96.4	2.0	1.2–3.2	339	278 (82.0)	77.5–85.9	1.3	0.9–1.7	321	237 (73.8)	68.7–78.6	1.3	1.0–1.7
40–64	5,523	5,494	5,251 (95.6)	95.0–96.1	2.6	2.2–3.2	5,380	4,836 (89.9)	89.1–90.7	2.5	2.1–2.9	5,217	4,389 (84.1)	83.1–85.1	2.4	2.1–2.8
65–74	2,663	2,635	2,522 (95.7)	94.9–96.5	2.7	2.2–3.5	2,530	2,264 (89.5)	88.2–90.7	2.4	2.0–2.8	2,376	2,009 (84.6)	83.0–86.0	2.5	2.1–3.0
75–	1,887	1,805	1,608 (89.1)	87.6–90.5	1	-	1,600	1,251 (78.2)	76.1–80.2	1	-	1,357	927 (68.3)	65.8–70.8	1	-

The highest (96.2, 90.5 and 86.2%) and lowest (93.3, 85.6 and 78.3%) 1-, 3- and 5-year age-adjusted adherence rates were found in the South HCR and in the West HCR, respectively (Table 3). The age-adjusted adherence rate to AET in the South-East, and North HCRs was significantly higher than in the West HCR at 3- and 5-year FU, but not at 1-year FU. In the HCR of Stockholm/Gotland and Uppsala/Örebro, no significant difference was seen in adherence compared to the West HCR. Adjustment also for T and N did not affect the result.

**Table 3:** Likelihood of adherence to adjuvant endocrine treatment at 1-, 3- and 5-year follow up after initiated treatment by public health care region, municipality (main groups) and presence of oncologic clinic in the region*. Logistic regression analysis (Odds ratio (OR) and 95% Confidence interval (CI)) adjusted for age at diagnosis.

	Adherence 1-year follow-up					Adherence 3-year follow-up					Adherence 5 year follow-up				
	Mean age at diagnosis, years (range)	Yes		Binary bivariate logistic regression		Mean age at diagnosis, years (range)	Yes		Binary bivariate logistic regression		Mean age at diagnosis, years (range)	Yes		Binary bivariate logistic regression	
		*N* (%)	95 % CI	OR	95% CI		*n*	95% CI	OR	95% CI		*n*	95% CI	OR	95% CI
Total	61.6 (23–101)	9,707 (94.4)	94.0–94.9			61.6 (23–101)	8,625 (87.7)	87.0–88.3			61.1 (23–97)	7,559 (81.7)	80.9–82.4		
Health Care Region (total *n* = 6)															
West	62.0 (27–92)	1,627 (93.3)	92.1–94.5	1	-	62.0 (27–92)	1,415 (85.6)	83.8–87.2	1	-	61.4 (27–92)	1,211 (78.3)	76.2–80.4	1	-
Stockholm-Gotland	60.5 (23–92)	2,188 (93.7)	92.7–94.7	1.0	0.80–1.3	60.5 (23–92)	1,952 (86.7)	85.2–88.1	1.1	0.90–1.3	60.0 (23–92)	1,714 (79.9)	78.2–81.6	1.1	0.93–1.3
Uppsala-Örebro	62.3 (28–97)	2,229 (94.4)	93.4–95.3	1.2	0.94–1.6	62.3 (28–97)	1,970 (87.1)	85.6–88.4	1.2	0.95–1.4	61.7 (28–97)	1,692 (80.0)	78.2–81.6	1.1	0.94–1.3
South-East	61.6 (25–92)	1,021 (94.3)	92.7–95.6	1.2	0.8–1.6	61.6 (25–92)	917 (88.7)	86.6–90.6	1.3	1.0–1.7	61.1 (25–92)	820 (84.2)	81.7–86.4	1.5	1.2–1.8
South	61.9 (25–92)	1,884 (96.2)	95.3–97.0	1.8	1.4–2.5	61.9 (25–92)	1,688 (90.5)	89.1–91.8	1.6	1.3–2.0	61.4 (25–92)	1,517 (86.2)	84.5–87.8	1.7	1.4–2.1
North	61.7 (24–101)	758 (94.8)	93.0–96.2	1.3	0.89–1.8	61.7 (24–101)	683 (88.7)	86.3–90.9	1.3	1.0–1.7	61.0 (30–88)	605 (84.5)	81.6–87.1	1.5	1.2–1.9
Regions* without oncologic clinic, n6	66.8 (25–101)	1,854 (91.3)	90.0–92.5	1	-	65.5 (26–101)	1,317 (83.0)	81.1–84.9	1	-	64.2 (26–93)	1,078 (75.5)	73.2–77.8	1	-
Regions* with oncologic clinic, n15	60.9 (23–97)	7,854 (95.2)	94.7–95.7	1.7	1.4–2.0	60.9 (23–97)	7,308 (88.6)	87.9–89.3	1.5	1.3–1.7	60.5 (23–97)	6,481 (82.8)	81.9–83.6	1.5	1.3–1.7
Cities															
Large cities total (A)	61.1 (25–92)	3,470 (93.8)	92.9–94.5	1	-	61.1 (25–92)	3,083 (86.7)	85.6–87.0	1	-	60.6 (25–92)	2,682 (79.9)	78.5–81.3	1	-
Medium-sized towns (B)	61.1 (23–101)	3,702 (94.8)	94.1–95.5	1.2	1.0–1.5	61.1 (23–101)	3,286 (88.3)	87.2–89.3	1.2	1.0–1.3	60.6 (23–97)	2,892 (82.5)	81.2–83.7	1.2	1.0–1.3
Small towns (C)	63.1 (30–93)	2,535 (94.8)	93.9–95.6	1.3	1.0–1.6	63.1 (30–93)	2,256 (88.1)	86.8–89.3	1.2	1.0–1.4	62.5 (30–93)	1,985 (82.9)	81.3–84.4	1.2	1.1–1.4

* = region (former counties), tot *n* = 21.

Women diagnosed in regions with an oncologic clinic (*n* = 15) had a significantly higher age-adjusted adherence compared to women diagnosed in regions without an oncologic clinic (*n* = 6) at 1-, 3- and 5-year follow-up (95.2% vs. 91.3%, 88.6% vs. 83.0% and 82.8% vs. 75.5%, respectively). Mean age in regions with an oncologic clinic was lower than in regions without.

Among the municipality groups, age-adjusted adherence was significantly lower in large cities (group A): (93.8, 86.7 and 79.9% at 1-, 3- and 5 year FU, respectively) compared to medium sized (group B) and small towns (group C) (94.8 and 88.3%, 82.5 and 94.8%, 88.1 and 82.9%, respectively). Mean age at diagnosis in the three large municipality group (A, B and C) was highest in the smallest towns (C). The adherence to AET was significantly higher in medium-sized and small towns than in large cities (Table 3), but the difference was small.

The 1-, 3- and 5-year adherence was highest in the age group included in the service screening programme with mammography (95.6, 89.9 and 84.1% for women 40–64 years and 95.7, 89.5 and 84.6%, respectively, for women 65–74 years) and considerably lower in women outside the screening programme (< 40 years: 94.2, 82.0 and 73.8% and ≥ 75 years: 89.1, 78.2 and 68.3%). Women younger than 40 years had significantly better adherence than the elderly, especially the first year after first expedition of AET (Table 2). Only women < 40 years at 5-year follow-up and women ≥ 75 years at 3- and 5-year follow-up had an adherence rate below the cut-off (80%).

## Discussion

This study investigated AET adherence in patients with early BC in Sweden with focus on regional differences. Swedish national guidelines include recommendations concerning AET after early ER+ BC and are used at all BC centres throughout the country. Nevertheless, the main finding of this study was that adherence differed significantly between HCRs. At the 5-year follow-up, adherence was significantly lower in large cities than in mid-sized and smaller towns. Moreover, we also found that adherence differed significantly between age groups, which is consistent with results from previous studies.

Two strengths of this study were the large study population and the high coverage rate (>95%) in the three registries that constituted the base of this study.

In comparison to previous international studies, the overall adherence in Sweden should be considered high [7, 10, 16–20]. In the recent study of adherence in Jönköping, there was a maintained high adherence at 5 year FU. In that study, adherence was even better at 5 year FU, whereas it decreased over time in this study [12]. In this study, patients who were non-adherent at 3 years were included from the 5-year analysis but excluded in the study from Jönköping. When including the 3-year-non-adherers at 5-year FU, there is a possibility that one individual with 75% MPR at 3-year FU will reach 85% adherence at 5-year FU due to increased adherence in the last 2 years (year 4–5). Adherence for this individual will thereby change ‘adherence-status’ from non-adherent at 3-year FU to adherent at 5-year FU. When comparing adherence between studies, it is therefore important to pay attention to the method used. This difference at 5 year FU between these two studies from Sweden may be explained by different methods used to calculate adherence at 5 years.

Czene et al. reported an adherence rate lower than that reported in this study. One explanation for the difference may be that patients in that study were censored if 180 days or more elapsed between dispensations. In this study, all dispensations were included, regardless of the time elapsed between dispensed doses. We believe that many women on AET take shorter and sometimes longer breaks from intake without planning to permanently stop the adjuvant treatment. These breaks are, we believe, usually due to side effects that can interfere with factors such as special occasions at work, family life and – last, but not least – sexual life and partnership [5, 6].

**Figure 2:**
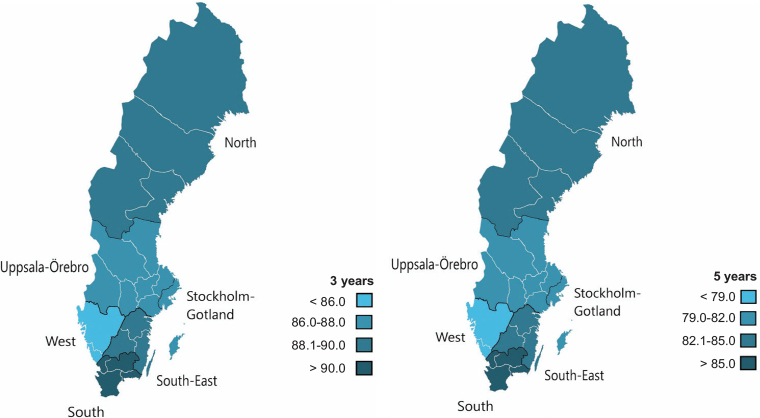
Map over Sweden showing adjusted adherence rate at 3- and 5-year follow-up in the 6 different Health care regions.

**Figure 3:**
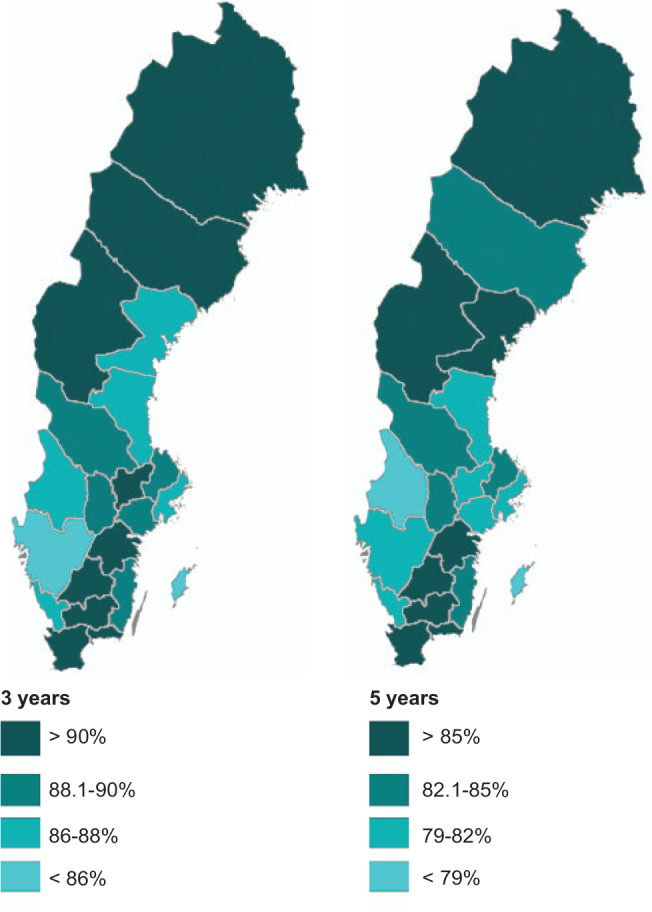
Map over Sweden showing adjusted adherence rate at 3- and 5-year follow-up in the 21 different regions.

There were noticeable differences in AET adherence within the country (Table 3, Figures 2 and 3) in this study. Median age and age distribution could be considered as confounding factors when studying adherence. The calculations were therefore adjusted for age, even though median age and age distribution was comparable between the HCRs.

The highest and lowest adherence rates were observed in the South and West HCR, respectively, at 1-, 3- and 5-year FU.

In this study, the west HCR had the lowest adherence, where also the lowest 10-year breast cancer specific survival among the studied population was noticed (data from National Quality Register for Breast Cancer) [21]. The south HCR had the highest adherence and correspondingly also the highest 10-year survival. This was an interesting finding, but the pattern was not consistent in all HCRs, why one should be careful to draw any conclusions and further studies are needed.

Adherence also varied by municipality groups, in the main groups (group A, B and C) (Table 3). At 5 years, the main groups showed a significantly lower adherence in large cities (group A), but the numbers were small, this is why the result must be interpreted with caution. As one of the three large cities is located in the west region where we found the lowest adherence, there is a possibility that this difference was related to the HCR and not to the city size. Median age was comparable in all groups (median age 61–63 years).

A previous qualitative study compared women with BC in northern Sweden and Stockholm County. That study showed that women in the north received most of their knowledge on why they were prescribed AET from their health care provider, whereas patients in Stockholm received information from several internet sources, as well as from their health care provider [22]. This could indicate that health care provider at hospitals in different HCRs and regions might give different information to their patients or use different methods to inform patients. The use of electronic support systems for breast cancer patients, for example, digital care plan, could increase the possibility to get equivalent information despite place of residence. This system was not available at the time of this study.

Treatment recommendations for the studied cohort are the same in all HCRs and regions due to national guidelines. Since there are some regional interpretations of these guidelines, the local variations in FU routines and patient information, might influence adherence. Patient information about the treatment is crucial, as patients might grow less motivated to continue treatment due to side effects in combination with sparse information.

Regions in the same HCR should have similar routines, since the routines are often set at the HCR level. However, adherence was found to differ between regions within the same HCR, which could indicate that there were local differences in routines even within HCR, which might depend on presence of oncologic clinic in the region, social factors or other unknown factors. The use of other health care providers than oncologists such as general practitioner (GP) to manage AET after breast cancer might vary between HCR and regions. Whether this affects adherence is not easy to study, since it probably differs between health care centres and even between the different GP. Adherence was found to be higher in regions with oncologic clinic compared to regions without. In regions without oncologic clinic, treatment, FU and side-effects often is handled by physicians at another clinic than oncology, for example, by surgeons or GP, where physicians and nurses may have less knowledge of the treatment recommendations and how to handle side effects. Most of these regions cooperates with oncologic clinics in regions nearby, providing them with consulting oncologists, but usually not on every-day basis. There is an ongoing effort to create oncologic clinics in these regions, but the need of support from GPs and surgeons in handling AET will probably remain. Other factors such as socio-economic factors are also well-known factors, which can affect adherence. No data were available concerning socioeconomic status, which could have provided further knowledge about differences in adherence. In Sweden, the government covers almost all treatment expenses, meaning that non-adherence should seldom depend on whether a patient can afford the treatment or not.

In line with the findings of other studies, adherence to AET was lower in younger (< 40 years) and older (≥ 75 years) women than in those aged 40–74 years [10, 18, 23]. The younger women had good adherence the first year but at 3- and 5-year FU, the adherence was significantly lower than for the group 40–74 years.

In elderly patients, quality of life during their remaining years might be affected by the adverse effects of AET and one can speculate if this could decrease adherence [24]. Thus, comorbidity and polypharmacy should be considered in elderly women especially and the benefits of AET carefully evaluated.

However, if indicated, AET should be started, due to benefits in all age groups. Close monitoring of adverse effects may increase adherence and decrease discontinuation due to side effects. For young women treated for BC, the duration of AET is a complicating factor in many aspects, such as conflict with pregnancy desire, impaired sexual function, which can affect close relationships, and other side effects affecting quality of life. GnRH is sometimes a part of AET in younger women and can affect adherence in this group. In comparison to TAM, the addition of GnRH to the treatment can have more severe adverse effects due to ovarian suppression and decreased oestrogen levels. The GnRH treatment is given as a subcutaneous injection and is sometimes provided by the treating clinic in Sweden, meaning that GnRH treatment in that case was not included in the SPR. The effect of GnRH on AET adherence in younger women could therefore not be investigated in this study. In the group of women aged 40–74, the same factors, as for younger and older women, can affect the adherence but maybe not as pronounced and hereby also show better adherence.

Since all treatment data were collected from a register, we cannot be sure that every patient took the drugs dispensed. Using MPR as method for calculating adherence assumes that the patients take their medicine. However, the number of patients who over the course of 5 years had the prescribed drug dispensed without taking it was probably small and unlikely to affect the study result. We did not censor any delivered doses in any prescription if they were overlapping in days with next expedition since we believe that this could be expected, otherwise patient could risk running out of medicine.

The fact that patients with previous cancer other than breast cancer were included in the study might affect adherence for some patients in both increased and decreased adherence. Some patients are not willing to be exposed to anti-cancer-drugs with side effects one more time, while other are willing to accept all treatments. This could be a limitation of the study.

Another limitation of this study was that no data concerning reason for discontinuing AET was available in the register.

In conclusion, adherence to AET in BC patients in Sweden was high in this study compared with most other previous national and international studies. Varying adherence rates between HCRs were seen, and with adherence found to be slightly lower in urban areas. The same national guidelines on AET were used in all BC centres throughout the country, and still, we could find differences between the HCR. Since adherence is important to reduce mortality and BC recurrences, the aim should be to further increase adherence. To strive for 100% adherence is not reasonable since the side effects in both young, middle aged and older women sometimes justify discontinuation of treatment. The care provider should however provide support to the woman before the decision to end treatment with AET is made. More studies regarding regional differences in FU and patient information, in addition to confounders, for example, tumour and treatment characteristics, socioeconomic status and comorbidity, are needed to find strategies for increasing AET adherence after early ER-positive BC.

## Statement and Declarations

### Data availability

Due to regulatory restrictions, data from this study is not openly available but can be available from the corresponding author, upon reasonable request and additional ethical approval.
